# Endoscopic Observation of the Growth Process of a Right-Side Sessile Serrated Adenoma/Polyp with Cytological Dysplasia to an Invasive Submucosal Adenocarcinoma

**DOI:** 10.1155/2016/6576351

**Published:** 2016-06-29

**Authors:** Kaoru Omori, Kanako Yoshida, Sadafumi Tamiya, Tsutomu Daa, Masahiro Kan

**Affiliations:** ^1^Department of Gastroenterology and Hepatology, Sato Daiichi Hospital, 77-1 Hokyoji, Usa, Oita 879-0454, Japan; ^2^Department of Pathology, Kitakyushu Medical Center, 2-1-1 Bashaku, Kitakyushu, Fukuoka 802-0077, Japan; ^3^Department of Pathology, Graduate School of Medicine, Faculty of Medicine, Oita University, 1-1 Idaigaoka, Yufu, Oita 879-5593, Japan

## Abstract

A sessile serrated adenoma/polyp (SSA/P) with cytological dysplasia in the right colon, which transformed to an invasive submucosal adenocarcinoma finally, was endoscopically observed in a 76-year-old woman. A whitish soft SSA/P (approximately 25 mm in diameter) was detected in the cecum. Biopsy samples were obtained from the small nodule, and the lesion was eventually diagnosed as an SSA/P with cytological dysplasia, considering endoscopic observations, among which the narrow-band imaging features suggested that the lesion was adenomatous, that is, a round-oval pattern, and hyperplastic, that is, comprising a circular pattern with dots and an invisible capillary vessel. After 11 months, an SSA/P had rapidly developed into a submucosal adenocarcinoma with lymphatic infiltrations, and the most aggressive deep invasion was observed in the central depression. This case suggests that right-side SSA/Ps with cytological dysplasia should be removed immediately, considering the potential for rapid progression to a larger size and eventually to deep and extensive cancer.

## 1. Introduction

According to the traditional theory of colorectal carcinogenesis involving an adenoma-carcinoma sequence, adenoma is recognized as a precursor lesion of colorectal cancer. In recent years, serrated lesions, including hyperplastic polyps (HPs), traditional serrated adenomas, and sessile serrated adenomas/polyps (SSA/Ps), have been increasingly recognized as being precursor lesions of colorectal cancer [1]. Among these serrated lesions, SSA/Ps have unique characteristics with regard to their clinical and genetic background, such as a proximal dominant location, high frequency of BRAF mutations, and a CpG island methylator phenotype [2]. Therefore, SSA/Ps have been regarded as precursor lesions for colorectal cancer. Although SSA/Ps have been reported to show rapid growth to an early cancer [3], few reports have elucidated the rapid progression speed of SSA/Ps to a large size and associated morphological changes during the transition to an adenocarcinoma sequentially. Here we report a rare case of the endoscopic observation of colon cancer which transformed sequentially from a large right-side SSA/P with cytological dysplasia to an invasive submucosal adenocarcinoma with lymphatic infiltrations, and the most aggressive deep invasion was observed in the central depression.

## 2. Case Presentation

**Figure 1:**
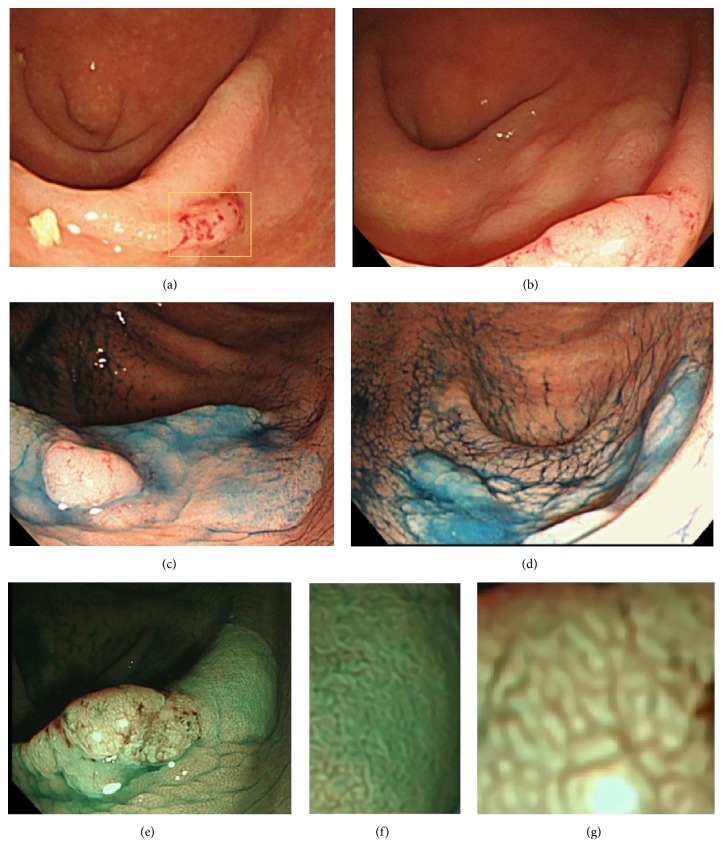
((a) and (b)) Conventional endoscopic view of a sessile serrated adenoma/polyp (SSA/P) in the cecum. The SSA/P was observed as a whitish laterally spreading soft lesion (approximately 25 mm in diameter) that harbored a small nodule with adhesion of some blood (approximately 5 mm in diameter). ((c) and (d)) Chromoendoscopic view of an SSA/P stained with indigo carmine. The edges and a small nodule of the lesion are emphasized. (e) Endoscopic view of an SSA/P with narrow-band imaging. (f) A pattern with a central dark area surrounded by a clear lighter area, so-called circular pattern with dots, was observed mostly. (g) A central light area surrounded by a dark outer area, so-called round-oval pattern, was observed in a small nodule.

**Figure 2:**
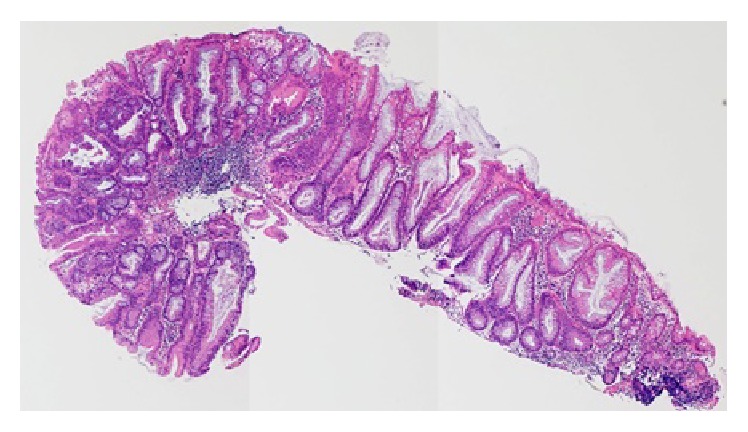
Pathological findings of the biopsy sample obtained from the small nodule (yellow box in Figure 1(a)). Hematoxylin-eosin staining of the sections of the biopsy sample revealed histologic features of a sessile serrated adenoma/polyp with cytological dysplasia at the curved portion (×40).

**Figure 3:**
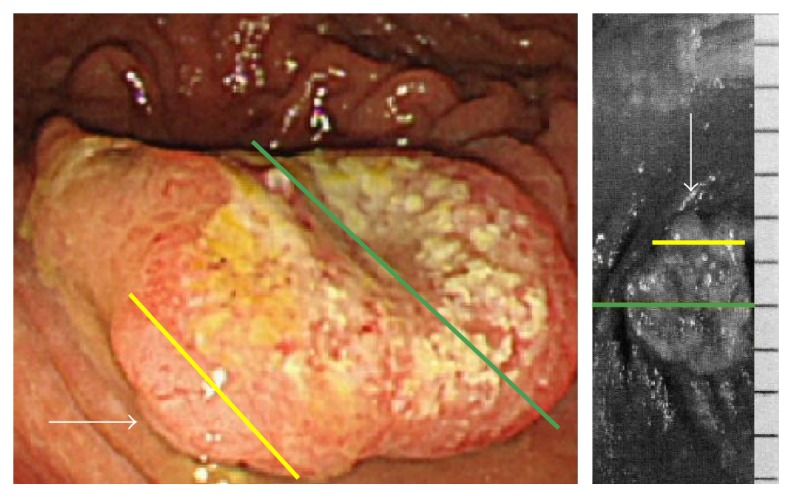
Conventional endoscopic view of a cancer arising from a sessile serrated adenoma/polyp. An elevated lesion with a central depression was observed at the same site in the cecum where a small nodule had been identified previously (white arrow).

**Figure 4:**
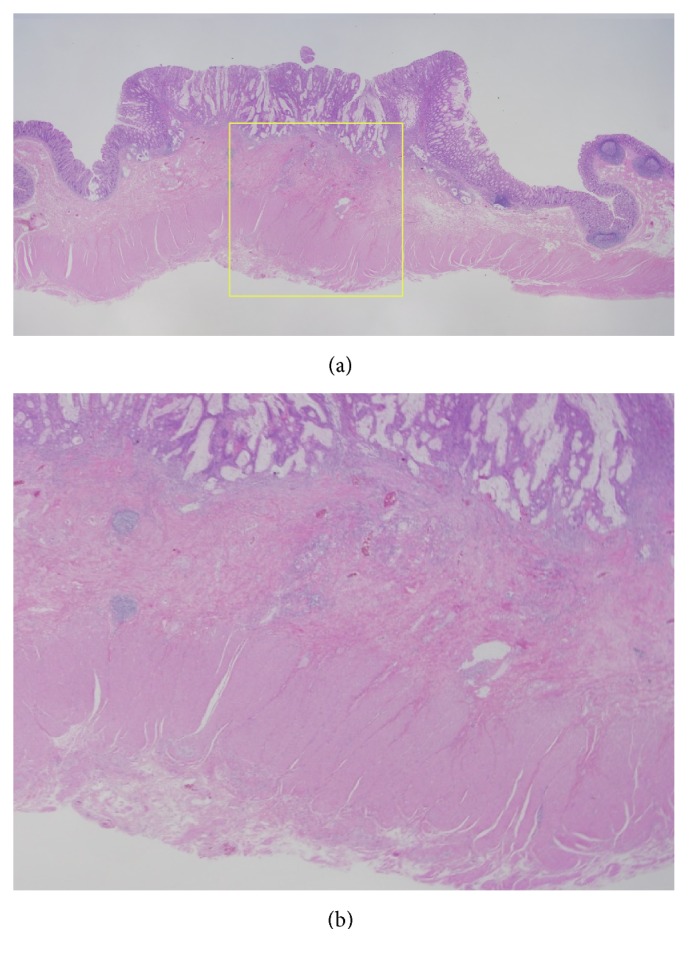
(a) Pathological findings of the resected lesion(approximately 40 mm in diameter). Hematoxylin-eosin staining of the sections of the resected specimen revealed a moderately differentiated adenocarcinoma that extended into the submucosal layer to a depth of 2700 *μ*m with mucin pools and lymphatic infiltrations (green line in Figure 3, ×4). (b) A magnified view of the most aggressive deep infiltration site of submucosal invasion (yellow box in (a), ×40).

**Figure 5:**
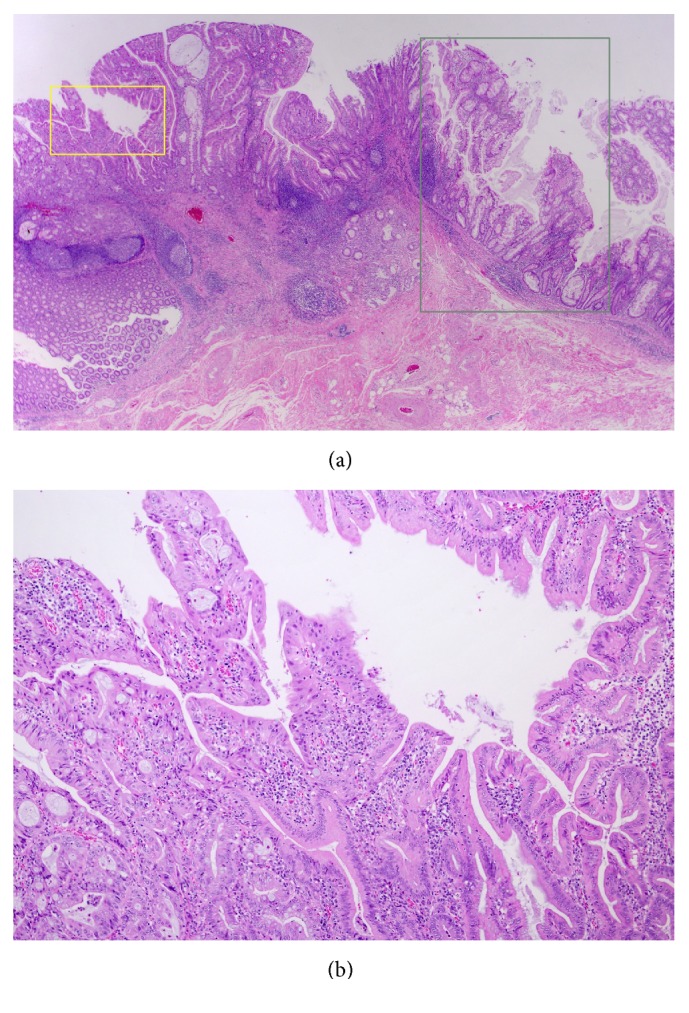
(a) A component of the sessile serrated adenoma/polyp (SSA/P) remained at the periphery of the invasive cancer (yellow line in Figure 3, ×12.5). The green box indicated a component of the SSA/P without cytological dysplasia. (b) A magnified view of a yellow box in (a). Histologically, a transition from the SSA/P with cytological dysplasia to a moderately differentiated adenocarcinoma was identified (×200).

A 76-year-old woman with diabetes mellitus visited our hospital for routine follow-up. Physical examination revealed no abnormalities. Except for her glucose metabolism, no abnormalities were observed on routine laboratory examinations, including blood biochemistry for serum tumor markers such as carcinoembryonic antigen and carbohydrate antigen 19-9. A laterally spreading soft lesion was detected in the cecum (Figures 1(a)–1(d)). Observation using colonoscopy showed a small nodule with adhesion of some blood (approximately 5 mm in diameter) and a whitish laterally spreading soft lesion (approximately 25 mm in diameter) (Figures 1(a)–1(d)). On narrow-band imaging (NBI), a pattern with a central dark area surrounded by a clear lighter area, that is, a circular pattern with dots, and an invisible capillary vessel, were observed as hyperplastic features, whereas a central light area surrounded by a dark outer area, that is, a round-oval pattern, was observed as an adenomatous feature in a small nodule (Figures 1(e)–1(g)). Therefore, biopsy samples were obtained from the nodule presenting with an adenomatous feature (Figure 2), and the lesion was eventually diagnosed as an SSA/P with cytological dysplasia, considering the endoscopic observations including the NBI features. The potential risk of progression to cancer was explained to the patient, because of the SSA/P features, that is, location in a right-side colon, being of large size, and being with cytological dysplasia. Endoscopic submucosal dissection was thought to be feasible but was not performed at the patient's request. After 11 months, follow-up colonoscopy was performed. At the same site in the cecum, an elevated lesion with a central depression was observed, where a small nodule had been identified earlier on the basis of the similar appearance and location of the nodule in both the cancer and SSA/P (Figure 3). Biopsy samples were obtained from the central depression site, and the elevated lesion was eventually diagnosed as a moderately differentiated adenocarcinoma. Based on these endoscopic findings, an invasive adenocarcinoma arising from the SSA/P was suspected. Endoscopic resection could not be performed and the lesion was removed surgically. The pathological diagnosis was a moderately differentiated adenocarcinoma extending 2700 *μ*m into the submucosal layer with a mucin pool, lymphatic infiltration (Figures 4(a) and 4(b)), in which the most aggressive deep invasion was observed in a central depression (Figures 4(a) and 4(b)), and a remaining SSA/P with/without cytological dysplasia component that was detected at the periphery of the invasive cancer (Figures 5(a) and 5(b)). Neither recurrence nor metastasis of colon cancer has been detected in the patient till now.

## 3. Discussion

We report a rare case of an SSA/P with cytological dysplasia progressing to an invasive submucosal adenocarcinoma with lymphatic infiltrations accompanied by distinct morphological changes as observed endoscopically. This case provides information on the risk of malignant transformation of an SSA/P with cytological dysplasia and its management in the right colon. Serrated lesions, including HPs, traditional serrated adenomas, and SSA/Ps, are precursor lesions in the new carcinogenesis pathway for colorectal cancer [1]. SSA/Ps located in the right colon have been considered to be high-risk lesions for progression to colorectal cancer [4]; however, they are sometimes difficult to identify and diagnose correctly by endoscopy because of their flat growth pattern, fuzzy appearance, and presence of abundant mucous [5]. Unmagnified NBI endoscopy enables better visualization of enhanced pit and capillary patterns, which showed fairly good agreement for the high accuracy of real-time histology prediction [6, 7]. Because magnified NBI endoscopy is not widely used, the findings of unmagnified NBI endoscopy are always important, similar to the usefulness of magnified NBI endoscopy. The lesion was eventually diagnosed as an SSA/P with cytological dysplasia, considering the pathological findings of the biopsy samples and the endoscopic observations, among which the NBI features suggested that the lesion was adenomatous, that is, a round-oval pattern, and hyperplastic, that is, comprising a circular pattern with dots and an invisible capillary vessel [6, 7]. The cancer was found at the same site 11 months later, arising from the SSA/P; the NBI feature of adenocarcinoma has been reported to show an irregular pattern [6]. Histologically, the resected lesion was finally diagnosed as a moderately differentiated adenocarcinoma extending 2700 *μ*m into the submucosal layer with lymphatic infiltrations. SSA/Ps are believed to become serrated pathway cancers via a series of molecular alternations, including BRAF mutation and CpG island methylation, with epigenetic inactivation of the mismatch repair gene MLH1 resulting in microsatellite instability [5]. The first question is why such molecular alternations and transitions to colorectal cancer happened rapidly. The characteristicsof serrated lesions require further research. Although there has been a significant reduction in the incidence of colorectal cancer in the left colon, the incidence and mortality rates for colorectal cancer in the right colon have not decreased in recent years [8]. It is very likely that a significant proportion of these cancers evolve from SSA/Ps that are undetectable during primary colonoscopy [9]. It is believed that up to 20% of all colorectal cancers arise through a serrated lesion pathway [10]. As observed in the present case, where an SSA/P presents a high risk of growing larger relatively quickly and rapidly progressing to an invasive cancer with deep infiltrations within 11 months, although it was previously reported that the progression rate of SSA/P-derived cancers was likely to be variable, some are rapid and some are gradual [11]. To reduce the incidence of colorectal cancers in the right colon, gastroenterologists need to recognize and possibly remove SSA/Ps with cytological dysplasia immediately, considering the potential for rapid progression to a larger size and eventually to deep extensive cancer. As observed in our case, the small nodule of the SSA/P was considered to preserve after progression to an invasive carcinoma on the basis of the similar appearance and location of the nodule in both the cancer and SSA/P. On the other hand, the most aggressive deep infiltration was observed in the central depression of the cancer. Thus, the progression to the invasive cancer may occur in the center of the SSA/P. Considering a previous report, the center in SSA/Ps may always be the most aggressive deep infiltration site, when it transforms to cancer [3]. It was reported that the very limited intramucosal carcinoma arising in an SSA/P showed deep invasion from crypt bases [11]. Thus, the cancer arising in SSA/Ps may be characterized by deep invasion from crypt bases, not by permeation to the surface, resulting in the difficulty of early detection of abnormal endoscopic findings. Therefore, leaving a right-side SSA/P with cytological dysplasia untreated for several months is dangerous in SSA/P-derived cancers. If a right-side SSA/P with cytological dysplasia is not immediately removed, it should certainly be removed within a short period. On the other hand, it is thought that many SSA/Ps do not have the potential risk of invasive cancer progression. To facilitate a precise therapeutic approach, accumulation of additional detailed cases is necessary to further clarify the clinical characteristics of SSA/Ps with the potential risk of invasive cancer progression.
